# Lung Ultrasound Elastography by SWE2D and “Fibrosis-like” Computed Tomography Signs after COVID-19 Pneumonia: A Follow-Up Study

**DOI:** 10.3390/jcm12247515

**Published:** 2023-12-05

**Authors:** Carlos Paredes-Manjarrez, Francisco J. Avelar-Garnica, Andres Tlacaelel Balderas-Chairéz, Jorge Arellano-Sotelo, Ricardo Córdova-Ramírez, Eliseo Espinosa-Poblano, Alejandro González-Ruíz, Juan Carlos Anda-Garay, José Adan Miguel-Puga, Kathrine Jáuregui-Renaud

**Affiliations:** 1 Departamento de Imagenología, Hospital de Especialidades del Centro Médico Nacional Siglo XXI, Instituto Mexicano del Seguro Social, Ciudad de México 06720, Mexico; pancreaman@gmail.com (C.P.-M.); anndresb@yahoo.com (A.T.B.-C.); jarellanito96@hotmail.com (J.A.-S.); ric.cordova.rx@gmail.com (R.C.-R.); 2 Departamento de Inhaloterapia y Neumología, Hospital de Especialidades del Centro Médico Nacional Siglo XXI, Instituto Mexicano del Seguro Social, Ciudad de México 06720, Mexico; gadaca1962@yahoo.com.mx (E.E.-P.); freud_233@hotmail.com (A.G.-R.); 3 Departamento de Medicina Interna, Hospital de Especialidades del Centro Médico Nacional Siglo XXI, Instituto Mexicano del Seguro Social, Ciudad de México 06720, Mexico; juan.andag@imss.gob.mx; 4 Unidad de Investigación Médica en Otoneurología, Instituto Mexicano del Seguro Social, Ciudad de México 06720, Mexico; adan.miguel@imss.gob.mx

**Keywords:** COVID-19 pneumonia, shear wave velocity, lung “fibrosis-like” signs

## Abstract

The aim of this study was to assess the shear wave velocity by LUS elastography (SWE2D) for the evaluation of superficial lung stiffness after COVID-19 pneumonia, according to “fibrosis-like” signs found by Computed Tomography (CT), considering the respiratory function. Seventy-nine adults participated in the study 42 to 353 days from symptom onset. Paired evaluations (SWE2D and CT) were performed along with the assessment of arterial blood gases and spirometry, three times with 100 days in between. During the follow-up and within each evaluation, the SWE2D velocity changed over time (MANOVA, *p* < 0.05) according to the extent of “fibrosis-like” CT signs by lung lobe (ANOVA, *p* < 0.05). The variability of the SWE2D velocity was consistently related to the first-second forced expiratory volume and the forced vital capacity (MANCOVA, *p* < 0.05), which changed over time with no change in blood gases. Covariance was also observed with age and patients’ body mass index, the time from symptom onset until hospital admission, and the history of diabetes in those who required intensive care during the acute phase (MANCOVA, *p* < 0.05). After COVID-19 pneumonia, SWE2D velocity can be related to the extent and regression of “fibrotic-like” involvement of the lung lobes, and it could be a complementary tool in the follow-up after COVID-19 pneumonia.

## 1. Introduction

Lung autopsies from deceased patients with COVID-19 pneumonia have shown signs of pro-fibrotic disease, and indicators of multiple regenerative strategies are invoked to re-establish alveolar epithelial cells lost to infection [1]; in patients with long duration of the disease, the expression of pro-fibrotic markers is related to interstitial fibrosis [2]. However, the recovery from COVID-19 pneumonia is variable with varying lung abnormalities. After one year from disease onset, a meta-analysis of 14 studies (*n* = 1854 adults) showed a wide prevalence range of lung abnormalities between 7.1% and 96.7% [3]. Given the scale of the pandemic, assessments of the lung sequelae are a matter of interest for public health.

During the COVID-19 pandemic, Computed Tomography (CT) has been the gold standard for the diagnosis and follow-up of lung disease [4,5,6]. Ground-glass opacities are common findings, which could reflect inflammatory abnormalities or could be related to immature fibrosis; however, the significance of “fibrotic-like” signs is yet unclear [7]. In 42% of 52 patients with mild to severe disease, a three-month follow-up showed residual CT abnormalities including ground-glass opacities, with/without sub-pleural parenchymal bands [8]. After six months of follow-up, in 12 patients (6/12 with invasive mechanical ventilation), CT imaging showed interstitial changes and pulmonary fibrosis occurring in the same thoracic areas as those observed during the acute phase [9]; at a similar follow-up time, “fibrotic-like” changes were found in more than two-thirds of 118 patients with moderate or severe disease, which were mainly associated with old age and mechanical ventilation [10]. In survivors of severe disease, CT findings of lung fibrosis were associated with restrictive lung impairment [11,12]; nevertheless, intensive medical care for respiratory distress implies inherent risk factors for residual lung disease [13,14].

Lung ultrasonography (LUS), devoid of radiation and at a low cost, has also been used for the diagnosis and follow-up of patients with COVID-19 pneumonia [15,16,17]. After three months from hospital discharge, LUS has been able to discriminate mild interstitial lung disease in patients who had severe COVID-19 pneumonia [16]. Moreover, LUS shear wave elastography may allow the assessment of soft tissue elasticity by utilizing a dynamic stress to generate a shear wave to quantitatively assess tissue stiffness in terms of shear modulus or Young’s modulus [18]. However, an assessment of shear wave velocity on four thoracic areas (two anterior and two posterior) showed no conclusive results after three months of hospitalization due to COVID-19 pneumonia [19]. A non-significant tendency towards higher velocity was observed among the patients who developed interstitial lung disease, which was evaluated by CT in 84 of 108 patients, only after they presented other indicators of lung disease [19]. The assessment of only four thoracic areas and the performance of CT imaging just in patients with other indicators of lung disease may have limited the appraisal of shear wave elastography. Compared to healthy controls, patients with interstitial lung disease may show an increased velocity of lung surface waves [20].

This study was designed to assess shear wave velocity by LUS elastography (SWE2D) on 14 thoracic areas for the evaluation of superficial lung stiffness after COVID-19 pneumonia, according to the evidence of CT “fibrosis-like” signs in lung lobes, three times with 100 days in between, considering the respiratory function. The study included patients discharged from the hospital during four contagion waves, after moderate to severe disease with no further need for oxygen supply and a wide range of recovery times since disease onset.

## 2. Materials and Methods

### 2.1. Patients

**Figure 1:**
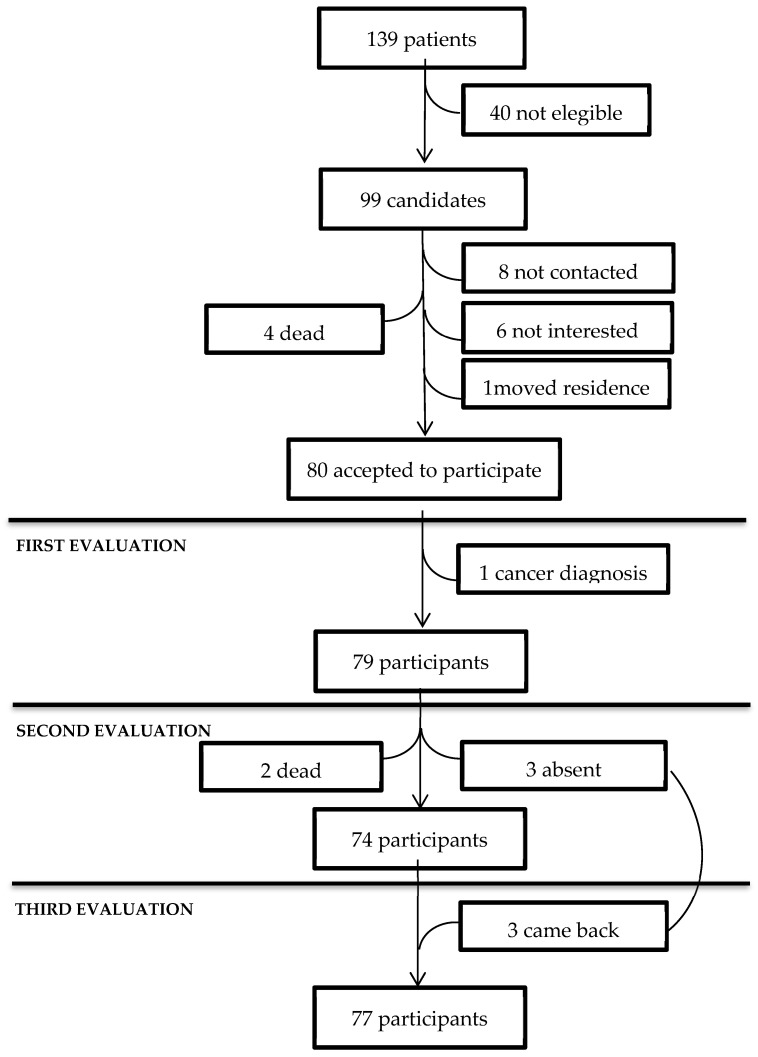
Flow chart of participants included in the study.

From May 2020 to May 2022, 139 patients with no need for oxygen supply were discharged from a single hospital. After the research protocol was approved by the local Research and Ethics Committees (R-2020-785-157), according to their clinical records, they were evaluated by the Internal Medicine Department to identify 99 patients with no history of lung disease, HIV, rheumatic disease, or contraindications for respiratory function testing [21]. Among ninety-one patients who were contacted, one moved their residency, four died, six denied participation, and eighty patients gave their informed consent to participate in the study; however, one patient abandoned the study due to a cancer diagnosis (Figure 1). Then, 79 patients participated in the study after 42 to 353 days from the onset of symptoms related to COVID-19 pneumonia and a hospital stay from 1 to 61 days (11.3 ± 10.8). During the acute phase, all the participants received an institutional standardized treatment during their hospital stay, when 14 of them were admitted to the Intensive Care Unit.

The participants were 30 females and 49 males, with a mean age of 52.3 years (±13.9 years). During follow-up, at the second evaluation, two patients had died, and three patients did not attend the appointment due to personal reasons (*n* = 74), but they came back for the third evaluation (*n* = 77) (Figure 1).

### 2.2. Procedures

After hospital discharge, paired evaluations (CT and LUS with elastography) were performed within the same day, three times with 100 days in between, along with an assessment of arterial blood gases and spirometry. The actual time of each of the three evaluations was 143 ± 70 days at the first evaluation, 242 ± 69 days at the second evaluation, and 340 ± 76 days at the third evaluation.

CT imaging was performed in the supine position, using a low-dose standard method (120 kVp with 50 mAs, range of 50 cm, and a collimated width of 0.1 mm) (Aquilion 64-slice; Toshiba, Tokyo, Japan). According to the Fleischner Society glossary of terms for thoracic imaging [22], findings considered to be “fibrosis-like” features included reticular opacities, inter-lobar and sub-pleural septal thickening, and sub-pleural bands. The evaluation of the images was performed by two independent senior radiologists who were unaware of the LUS observations; in case of discordance, another specialist performed a third revision. They subjectively estimated the extent of lobe involvement to indicate 0% for normal lobe parenchyma, <25% lobe involvement, 25% to 49% involvement, 50% to 75% involvement, and >75% involvement.

LUS on B mode with Shear Wave Elastography (SWE2D) (Logiq E9, General Electric; Boston, MA, USA), using a convex transducer (3–5 MHz; Abdominal 3.5C Logic 9, General Electric; Boston, MA, USA), was performed by a single experienced ultrasound specialist with the help of a single imaging resident to explore 14 areas (3 posterior, 2 lateral, and 2 anterior). During a single breath, the lungs were evaluated through the intercostal spaces on each side on the paravertebral line above the curtain sign, the paravertebral line at the inferior angle of the shoulder blade, the paravertebral line at the spine of the shoulder blade, the midaxillary line below the inter-nipple line, the midclavicular line below the inter-nipple line, and one intercostal space above the level of the diaphragm in the mid-axillary and mid-scapular lines, respectively. An elastogram was superimposed on a B-mode image and the shear wave velocity was measured. Images were stored (Enterprise-Imaging-Platform, Agfa-Health Care, Mortsel, Belgium), and they were evaluated off-line by two independent senior ultrasound medical specialists, blinded to the patient’s clinical condition; in the case of discordance, another specialist performed a third revision. LUS findings were classified according to Hasan et al. [23]: waves reflected by air (A-lines) or decreased gas–liquid ratio (B-lines). B lines were categorized according to the distance between them: if the distance was >7 mm (B7-lines), if it was 3 mm (B3-lines), or if the B-lines were confluent with ventilation decrease. After interpretation, the findings on each of the 14 thoracic areas were categorized according to their correspondence to each of the five lung lobes (right superior, middle, and inferior; and left superior and inferior); accordingly, the average shear wave velocity was estimated for each lung lobe. Difficulties in obtaining an appropriate signal on any of the 14 zones occurred in two to four patients per evaluation, on no more than two zones per patient.

Standard spirometry (EasyOne Air, ndd Medizintechnik AG, Zurich, Switzerland) was performed according to standard international recommendations [24] in order to assess forced expiratory volume in the first second (FEV1), forced vital capacity (FVC), FEV1/FVC ratio, 25%, 50%, and 75% forced expiratory flow (FEF), and peak expiratory flow (PEF). Before spirometry was performed, an arterial blood sample was obtained for assessment of arterial gases (Gem 5000 Premier, Instrumentation Laboratory, Werfen, Austria).

Statistical analysis was performed after a data distribution assessment with the Kolmogorov–Smirnov test. Accordingly, linear correlations were explored using the Pearson coefficient, where coefficients <0.30 were low, 0.3 to <0.5 were moderate, and >0.5 were high correlations [25]. Bivariate analysis was performed using *t*-tests, ANOVA, and repeated measures ANOVA, with Holm–Bonferroni corrections; the Tukey Honest Significance Difference was used as a post hoc test. Multivariate analysis was performed using MANCOVA. All tests were performed with a significance level of 0.05.

## 3. Results

### 3.1. Descriptive and Bivariate Analysis

#### 3.1.1. General Characteristics of the Participants

The general characteristics of the participants by gender are described in Table 1. Comparisons between females and males showed higher body mass index and higher frequency of diabetes in females than in men (*t*-test, *p* < 0.05).

#### 3.1.2. Computed Tomography

**Figure 2:**
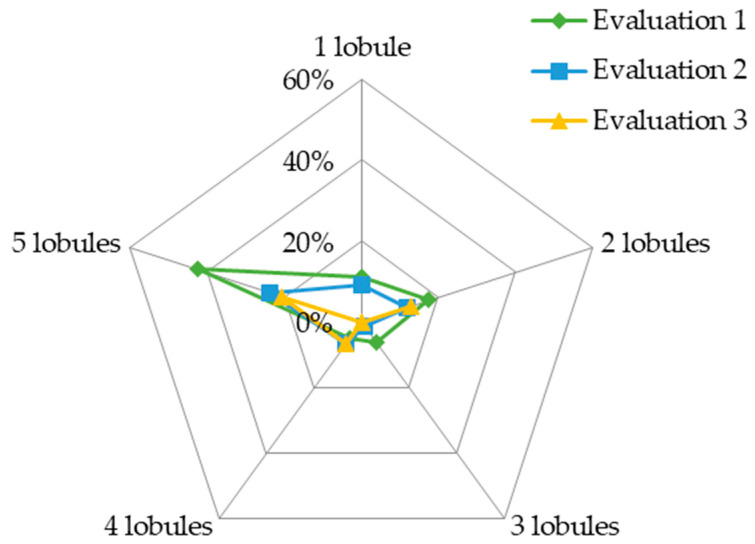
Reduction in the frequency of the number of lung lobes involved in each patient during each of the three evaluations by Computed Tomography.

**Figure 3:**
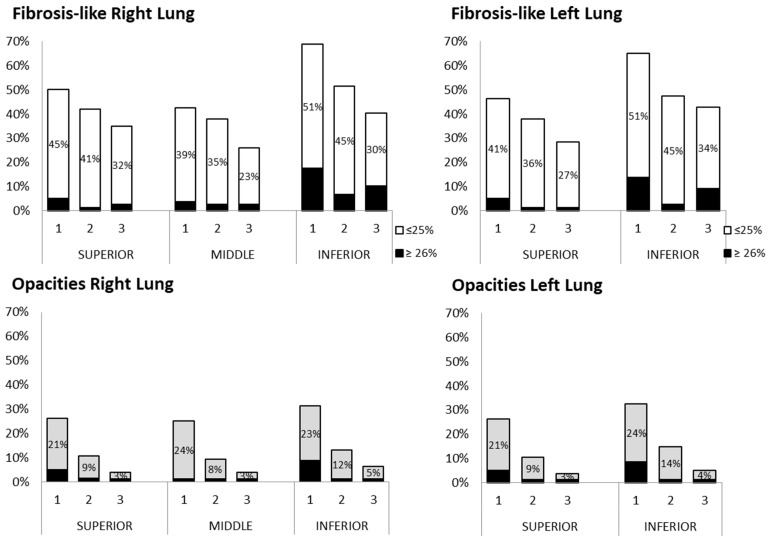
Frequency of “fibrosis-like” findings and ground-glass opacities according to lung-lobe involvement (25% or ≥26%) in the three evaluations (numbered 1, 2, and 3).

At the first evaluation, 77% of the patients (*n* = 61) showed abnormal CT signs; the number of lung lobes involved over time is depicted in Figure 2. At the first evaluation, involvement of all lung lobes was frequent; at that time, abnormalities were observed in the five lung lobes of 42.5% (*n* = 34) of the patients and decreased to 23.6% and 20.5% in the second and the third evaluations, respectively (Figure 2). However, lobe involvement was usually focal (<25%) and decreased over time (Figure 3). The most frequent findings were “fibrosis-like” signs and ground-glass opacities. In all evaluations, there were no signs of clustered cystic air spaces (honeycombing), air bronchogram, lymphadenopathy, or pleural effusion; although, at the first evaluation, one of the patients had <25% consolidation in the two inferior lobes, which was not evident at the following evaluations.

As the frequency of CT signs of lung abnormalities decreased, the frequency of no signs of disease increased as follows:

- At the first evaluation, 16 among 79 patients (20%) (eight females and eight males; 25 to 66 years old) showed no abnormalities; imaging was performed after 65 to 353 days from disease onset. They were admitted to hospital 2 to 20 days after disease onset; during the acute phase, two of them were admitted to the Intensive Care Unit.
- At the second evaluation, 30 among 74 patients (40%) (16 females and 14 males; 25 to 71 years old) showed no abnormalities; imaging was performed after 150 to 442 days from disease onset; the range of their clinical evolution from disease onset to hospital admission was the same; during the acute phase, three of them were admitted to the Intensive Care Unit.
- At the third evaluation, 36 among 77 patients (46%) (19 females and 17 males; 25 to 71 years old) showed no abnormalities; imaging was performed after 199 to 571 days from disease onset; the range of their clinical evolution from disease onset to hospital admission was the same; during the acute phase, five of them were admitted to the Intensive Care Unit.

#### 3.1.3. LUS with Elastography

Lung ultrasonography showed abnormalities in 34% (*n* = 27) of the patients at the first evaluation, in 16% (*n* = 12) of them at the second evaluation, and in 13% (*n* = 10) at the third evaluation. The common finding was B7-lines; just six patients showed B3-lines at the first evaluation, one of them died before the second evaluation, and, in the other five patients, this finding was not evident afterward. Table 2 shows the shear wave velocity measured by SWE2D in the three evaluations.

**Figure 4:**
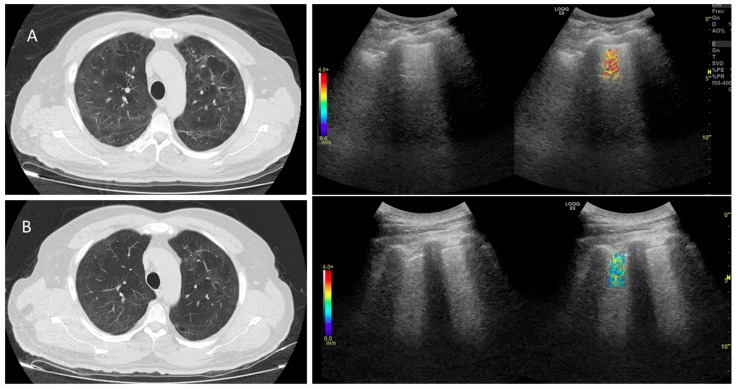
Ultrasonography on B mode, without/with elastography, and Computed Tomography images of a 62 y.o. male after 112 and 209 days since hospital discharge. The shear wave velocity measured on the left lung by SWE2D was 3.58 m/s in the first evaluation (**A**), and 2.20 m/s in the second evaluation (**B**). The decrease in the stiffness (color red in (**A**)) was consistent with a parallel decrease in the “fibrosis like” signs observed by Computed Tomography.

During the follow-up, the velocity decreased consistently, with significant differences between the first and the third evaluations for the five lung lobes (ANOVA and Tukey Honest Significance Difference, *p* ≤ 0.01). This decrease was consistent with the decrease in abnormal CT findings (Figure 4).

**Figure 5:**
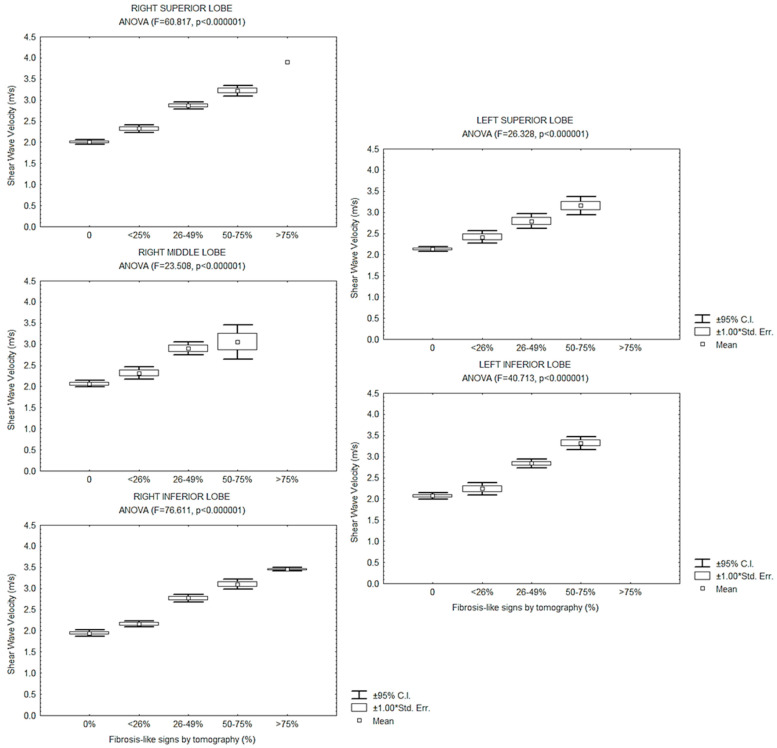
Mean, 95% Confidence Interval of the mean, and Standard Error of the mean (Std. Err.) of the shear wave velocity, according to lung lobe involvement by “fibrosis-like” findings observed by Computed Tomography, in all evaluations.

In each evaluation, the shear wave velocity measured by SWE2D changed according to the extent of the abnormal CT signs by lung lobe, with a lower velocity when the “fibrosis-like” involvement was less (ANOVA, *p* < 0.0001). The entire results by lung lobe are shown in Figure 5. Overall measurements were taken in the three evaluations, low to moderate linear correlations were observed between the shear wave velocity measured on each lung lobe with the age of the participants (r values from 0.24 to 0.38, *p* < 0.05), and with the time since disease onset until hospital admission (r values from 0.29 to 0.35, *p* < 0.05), with no evident correlations with the time of imaging since disease onset, or the time of hospital stay.

In addition, regardless of the sex, comparison between patients with/without body mass index (BMI) ≥ 30 kg/m^2^ (33/79 patients) showed that those with BMI ≥ 30 kg/m^2^ consistently exhibited lower shear wave velocity, particularly on the measurement of the left superior lobe (*t*-test, Holm–Bonferroni correction, *p* < 0.01).

#### 3.1.4. Respiratory Function Test

Table 3 shows the results of the three evaluations. During follow-up, the FVC and FEV1 increased proportionally (MANOVA, *p* < 0.005), with no change on the FVC/FEV1 ratio (*p* > 0.05), while the 25% FEF decreased (MANOVA, *p* < 0.05), with no significant change on the measurement of blood gases among evaluations (*p* > 0.05) (Table 3). However, in the three evaluations, both FVC and FEV1 were higher in men compared to women.

### 3.2. Multivariate Analysis

#### 3.2.1. General Covariates

The covariance analysis included the age and the BMI of the patients, their history of diabetes, the time from symptoms onset until hospital admission, the need for intensive care during hospital stay, and the time elapsed since disease onset until the first imaging evaluation (using a cutoff point of 6 months).

The variability of the shear wave velocity on each lung lobe was related to the age and the body mass index of the patients at hospital admission, the time from symptoms onset until hospital admission, and the interaction between the history of diabetes with the need for intensive care during the hospital stay (Table 4). The Multiple R estimated for these relationships ranged from 0.49 to 0.67 (*p* < 0.001), where the lower coefficients were those of the third evaluation. However, no influence of these variables was observed on the differences among the shear wave velocity measurements.

#### 3.2.2. Respiratory Function Covariates

At the first evaluation, the variance of the shear wave velocity on each lung lobe was related to FEV1 and FEF25%, considering sex, and with an inconsistent relation to arterial HCO3 (Table 5). The Multiple R values ranged from 0.43 to 0.59 (*p* < 0.005) for each of the five lung lobes. However, at the second evaluation, the only consistent relationship was with FEV1, considering sex (Table 5); yet the Multiple R value for each of the five lung lobes ranged from 0.38 to 0.56 (*p* < 0.03). However, at the third evaluation, these relationships were less consistent (Table 5), and a significant Multiple R value was evident just for the left inferior lung lobe (Multiple R = 0.40, *p* = 0.01).

## 4. Discussion

The main finding of this study is the agreement between the shear wave velocity (SWE2D) measurements and the extent of involvement of the lung lobes by CT findings at early and late evaluations after the acute stage of COVID-19 pneumonia, among patients and within the same patient. Consistently, the study also showed a parallel decrease in the shear wave velocity and abnormal CT signs.

The gradual resolution of the signs of lung damage is consistent with the report that survivors of COVID-19 pneumonia frequently improve and may recover after one year from the acute phase of the disease, although some patients may experience respiratory limitations, particularly those with severe disease and older age [26]. The CT findings were also consistent with the results of a meta-analysis of 11 studies showing that honeycombing is infrequent, while ground-glass opacities are the most frequently reported non-fibrotic change [3].

Lung ultrasound with elastography is an emerging imaging technology that can provide a quantitative assessment of tissue elasticity, including the lungs [18]. In this study, the shear wave velocity measurements consistently co-varied with the age and body mass index of the participants, as well as with the history of diabetes when intensive care was needed during hospitalization, and the time from disease onset until hospital admission. The relationship between age and body mass index is consistent with previous findings on the skin [27] and the pancreas [28]. Propagation of the shear wave in biological tissues is related to several factors, including the stiffness and density of the tissues and their intrinsic mechanical properties, which may change with age and body fat. Older age implies intrinsic changes as well as remodeling of tissues, including reduced lung elasticity [29,30], while fat may lead tissues to become softer, reducing shear wave velocity [28]. On the other hand, the clinical correlations observed in this study are consistent with previous studies showing that lung sequelae of COVID-19 pneumonia may be related to comorbidities and the need for intensive medical care [8]; in particular, diabetes mellitus has been recognized as a risk factor for lung fibrosis [31,32], while ventilation support implies inherent risk factors for residual lung disease [14]. Additionally, during the acute phase of COVID-19 pneumonia, the time of evolution of the symptoms before receiving medical care has been related to the extension of lung disease [33].

In each evaluation, the shear wave velocity was related to FEV1 and FVC. This finding is consistent with the relationship of these parameters with lung elasticity [29]. Even further, the gradual changes observed during follow-up suggest a gradual decrease in lung restriction over time. However, the results of the arterial gas evaluation showed limited oxygen saturation with no change among evaluations. Together, these results could be compatible with persistent limitations of gas exchange at the alveolar tissue level, which were not explored within the aims of this study. Albeit the findings of this study support the hypothesis of gradual resolution of lung inflammatory changes, the clinical benefit of anti-fibrotic and immune-modulatory therapies is yet unclear.

Previous evidence supports that pulmonary fibrotic sequelae after severe COVID-19 pneumonia can be detected by LUS [34,35]. In this study, we observed that LUS supplemented by SWE2D is related to the extent and regression of “fibrotic-like” involvement of the lung lobes after either moderate or severe COVID-19 pneumonia. However, further clinical research should be performed to translate these findings into clinical scenarios.

The main limitation of this study is the generalizability of the results. Since the study was designed to assess the use of shear wave velocity (SWE2D) for the evaluation of superficial lung stiffness in a selected group of patients recovering from COVID-19 pneumonia, participants were chosen after receiving standardized treatment in a single hospital, with no history of lung disease, rheumatic disease, HIV, or contraindications for lung function testing, and with no further need for oxygen supply after hospital discharge. Also, to assess the relationships among the study variables, all the assessments were recorded within a couple of hours in each of the three evaluations. Further studies in real clinical scenarios must be performed to assess the clinical application of lung shear wave velocity (SWE2D). The second limitation was the sample size, which did not allow the assessment of expected covariates of lung disease, such as tobacco use [36]. Another limitation was the lack of CO diffusion assessment for supplementary interpretation of the results, which had no implication in achieving the main goal of the study.

## 5. Conclusions

After COVID-19 pneumonia, shear wave velocity (SWE2D) measurements are consistent with the extent and regression of involvement of the lung lobes by abnormal CT signs and spirometry, regardless of the recovery time since disease onset, and within a range of circa 200 days follow-up. However, several factors showed an influence on lung shear wave velocity measurements, including the age and body mass index of the patients, as well as the history of diabetes with the need for intensive medical care during the acute phase of the disease.

## Figures and Tables

**Table 1:** General characteristics of the 79 participants of the study (S.D. for standard deviation). Comparisons between females and males were performed using *t*-tests, either for proportions or for means.

Variable	All (*n* = 79)		Females (*n* = 30)		Males (*n* = 49)		*p*
	Mean (S.D.)	Range	Mean (S.D.)	Range	Mean (S.D.)	Range	
Years of age	52.3 (13.9)	25–85	52.4 (12.2)	28–71	52.3 (15.0)	25–85	-
Body Mass Index at hospital admission	30 (5.4)	23.2–51	32.3 (6.6)	23.2–51	28.6 (4.0)	23.2–43	0.002
Days from symptom onset to hospitalization	9.3 (4.0)	1–22	8.1 (3.6)	1–15	9.9 (4.1)	2–22	-
Days in hospital	11.3 (10.8)	1–61	11.5 (12.8)	1–61	11.1 (9.4)	1–52	-
Oxygen saturation at hospital admission (%)	81.2 (11.6)	49–96	78.8 (12.6)	49–96	81.9 (11.0)	56–96	-
Oxygen saturation at hospital discharge (%)	93.8 (2.3)	88–98	93.4 (2.6)	88–98	94.0 (2.1)	90–98	-
	*n*	%	*n*	%	*n*	%	
Ever smokers	21	26.5%	5	16%	16	33%	
Systemic high blood pressure	29	36.7%	14	46%	15	31%	
Type 2 diabetes	32	40.5%	17	56%	15	30.6%	0.02
Intensive care during hospitalization	14	17.5%	6	20%	8	16%	-

**Table 2:** Mean and standard deviation of the mean (S.D.) of the LUS shear wave velocity of 79 patients at the first evaluation, 74 patients at the second evaluation, and 77 patients at the third evaluation.

Lung Lobe	Evaluation 1 (*n* = 79)	Evaluation 2 (*n* = 74)	Evaluation 3 (*n* = 77)	(F Value) *p* Value
	Mean (S.D.)	Mean (S.D.)	Mean (S.D.)	
Right superior	2.30 (0.47)	2.25 (0.43)	2.14 (0.47)	(4.86) <0.01
Right middle	2.31 (0.57)	2.29(0.49)	2.07 (0.61)	(6.33) <0.0001
Right inferior	2.40 (0.57)	2.24 (0.45)	2.10 (0.57)	(11.00) <0.0001
Left superior	2.34 (0.45)	2.18 (0.37)	2.13 (0.41)	(8.38) <0.0001
Left inferior	2.38 (0.61)	2.20 (0.48)	2.09 (057)	(10.26) <0.0001

**Table 3:** Mean and standard deviation of the mean (S.D.) of the respiratory function tests of 79 patients at the first evaluation, 74 patients at the second evaluation, and 77 patients at the third evaluation. Statistical significance ≤0.05 is highlighted by an asterisk (*).

Variable	Evaluation 1 (*n* = 79)	Evaluation 2 (*n* = 74)	Evaluation 3 (*n* = 77)	(F Value) *p* Value
	Mean (S.D.)	Mean (S.D.)	Mean (S.D.)	
**Spirometry**				
Forced vital capacity (FVC) (L)	3.5 (1.0)	3.6 (1.0)	3.6 (0.9)	(6.951) 0.001 *
1st sec forced expiratory volume (FEV1) (L)	2.8 (0.8)	3.0 (0.8)	3.0 (0.8)	(6.062) 0.002 *
FEV1/FVC ratio	82.1 (5.6)	82.2 (5.3)	82.3 (5.4)	-
Forced expiratory flow (FEF)				
25% (L)	6.3 (2.9)	5.8(2.4)	5.2 (1.8)	(5.974) 0.003 *
50% (L)	3.4 (2.0)	3.5 (1.3)	3.3 (1.2)	-
75% (L)	1.4 (1.7)	1.3 (0.4)	1.1 (0.3)	-
Peak expiratory flow (PEF) (L)	7.5 (2.8)	7.4 (2.4)	6.7 (1.7)	(3.658) 0.028
**Blood Gases**				
Partial pressure of carbon dioxide (mm Hg)	36.7 (3.2)	35.7 (3.0)	36.1 (3.4)	-
Partial pressure of oxygen (mm Hg)	62.5 (13.0)	64.8 (8.8)	66.1 (9.6)	-
Concentration of bicarbonate (mEq/L)	23.9 (1.7)	23.6 (1.6)	23.8 (1.1)	-
Oxygen saturation (%)	89.5 (10.5)	91.9 (4.6)	91.7 (4.6)	-
Relative excess or deficit of base (mmol/L)	−0.5 (1.8)	−0.7 (1.8)	−0.3 (1.5)	-
Lactate (mmol/L)	1.3 (0.6)	1.3 (0.5)	1.2 (0.5)	-

**Table 4:** F values and *p* values of the multivariate analysis of covariance according to general covariates of the LUS shear wave velocity measurements on each of the five lung lobes, in 79 patients at the first evaluation, 74 patients at the second evaluation, and 77 patients at the third evaluation. Statistical significance ≤0.05 is highlighted by an asterisk (*).

Lung Lobe	Right Superior		Right Middle		Right Inferior		Left Superior		Left Inferior	
Variable	F Value	*p* Value	F Value	*p* Value	F Value	*p* Value	F Value	*p* Value	F Value	*p* Value
Intercept	77.138	<0.00001	63.468	<0.00001	74.212	<0.00001	131.372	<0.00001	56.712	<0.00001
Age	7.653	0.007 *	4.390	0.040 *	4.846	0.031 *	2.14965	0.14758	2.98758	0.088
Body mass index at admission (BMI)	8.380	0.005 *	9.784	0.002 *	16.02	0.0001 *	16.921	0.0001 *	9.43672	0.003 *
Days until hospitalization (Time)	8.461	0.005 *	3.804	0.055	4.626	0.035 *	2.96932	0.089	9.524	0.003 *
>6 months after disease onset (6M)	3.055	0.085	4.376	0.040 *	2.288	0.135	0.975	0.327	1.177	0.282
Intensive care unit (ICU)	2.530	0.116	4.563	0.036 *	1.111	0.295	4.592	0.035 *	3.782	0.056
Diabetes (DM)	4.189	0.044 *	0.034	0.853	7.401	0.008 *	5.429	0.023 *	7.818	0.006 *
6M*UCI	4.866	0.031 *	2.472	0.120	0.844	0.361	2.438	0.123	0.990	0.323
6M*DM	1.201	0.277	1.192	0.279	1.1954	0.278	1.833	0.180	0.055	0.813
UCI*DM	14.604	0.0003 *	1.649	0.203	14.576	0.0003 *	10.93	0.001 *	9.237	0.003 *
6M*ICU*DM	3.183	0.079	3.230	0.077	3.232	0.076	5.329	0.024 *	2.142	0.14

**Table 5:** F values and *p* values of the multivariate analysis of covariance of the LUS shear wave velocity according to the respiratory function test on each of the five lung lobes, in the three evaluations, on 79 patients at the first evaluation, 74 patients at the second evaluation, and 77 patients at the third evaluation. Statistical significance ≤0.05 is highlighted by an asterisk (*).

Lung Lobe	Right Superior		Right Middle		Right Inferior		Left Superior		Left Inferior	
Variable	F Value	*p* Value	F Value	*p* Value	F Value	*p* Value	F Value	*p* Value	F Value	*p* Value
**Evaluation 1**										
Intercept	35.582	<0.0001 *	27.472	<0.0001 *	33.551	<0.0001 *	28.296	<0.0001 *	23.211	<0.0001 *
FEV1	18.048	<0.0001 *	12.296	0.001 *	19.999	<0.0001 *	11.866	0.001 *	24.020	<0.0001 *
FEF25	8.329	0.005 *	2.932	0.091	16.972	<0.0001 *	7.213	0.009 *	12.891	0.001 *
HCO3	4.041	0.048 *	3.825	0.054	5.060	0.027	1.775	0.187	1.816	0.182
SEX	6.948	0.010 *	3.408	0.069	15.407	<0.0001 *	14.540	<0.0001 *	11.020	0.001 *
**Evaluation 2**										
Intercept	28.471	<0.0001 *	21.759	<0.0001 *	23.956	<0.0001 *	35.952	<0.0001 *	43.686	<0.0001 *
FEV1	19.176	<0.0001 *	7.549	0.008 *	8.365	0.005 *	4.545	0.037 *	15.610	<0.0001 *
FEF25	3.300	0.074	1.106	0.297	1.276	0.263	0.015	0.904	1.536	0.219
HCO3	2.082	0.154	2.753	0.102	2.860	0.095	5.847	0.018 *	9.913	0.002 *
SEX	14.639	<0.0001 *	3.945	0.051	7.136	0.009 *	3.264	0.075	17.108	<0.0001 *
**Evaluation 3**										
Intercept	10.672	0.002 *	8.002	0.006 *	5.250	0.025 *	3.313	0.073	7.095	0.010 *
FEV1	4.514	0.037 *	2.945	0.091	3.692	0.059	1.779	0.187	7.785	0.007 *
FEF25	3.627	0.061	3.621	0.061	6.423	0.014 *	9.053	0.004 *	9.132	0.004 *
HCO3	1.731	0.193	1.607	0.209	0.587	0.446	0.036	0.851	1.001	0.321
SEX	2.908	0.093	0.930	0.338	3.175	0.079	0.806	0.372	3.792	0.056

## Data Availability

The data are contained within the article. The datasets are available from the corresponding author upon reasonable request.

## References

[1] Delorey T.M., Ziegler C.G.K., Heimberg G., Normand R., Yang Y., Segerstolpe Å., Abbondanza D., Fleming S.J., Subramanian A., Montoro D.T. (2021). COVID-19 tissue atlases reveal SARS-CoV-2 pathology and cellular targets. Nature.

[2] D’Agnillo F., Walters K.A., Xiao Y., Sheng Z.M., Scherler K., Park J., Gygli S., Rosas L.A., Sadtler K., Kalish H. (2021). Lung epithelial and endothelial damage, loss of tissue repair, inhibition of fibrinolysis, and cellular senescence in fatal COVID-19. Sci. Transl. Med..

[3] Bocchino M., Rea G., Capitelli L., Lieto R., Bruzzese D. (2023). Chest CT Lung Abnormalities 1 Year after COVID-19: A Systematic Review and Meta-Analysis. Radiology.

[4] Pan F., Ye T., Sun P., Gui S., Liang B., Li L., Zheng D., Wang J., Hesketh R.L., Yang L. (2020). Time course of lung changes at chest CT during recovery from coronavirus disease 2019 (COVID-19). Radiology.

[5] Rubin G.D., Ryerson C.J., Haramati L.B., Sverzellati N., Kanne J.P., Raoof S., Schluger N.W., Volpi A., Yim J.-J., Martin I.B.K. (2020). The Role of Chest Imaging in Patient Managemalest During the COVID-19 Pandemic. A Multinational Consensus Statemalest From the Fleischner Society. Chest.

[6] Solomon J.J., Heyman B., Ko J.P., Condos R., Lynch D.A. (2021). CT of Post-Acute Lung Complications of COVID-19. Radiology.

[7] Wells A.U., Devaraj A., Desai S.R. (2021). Interstitial Lung Disease after COVID-19 Infection: A Catalog of Uncertainties. Radiology.

[8] Tabatabaei S.M.H., Rajebi H., Moghaddas F., Ghasemiadl M., Talari H. (2020). Chest CT in COVID-19 pneumonia: What are the findings in mid-term follow-up?. Emerg. Radiol..

[9] Gulati A., Lakhani P. (2021). Interstitial lung abnormalities and pulmonary fibrosis in COVID-19 patients: A short-term follow-up case series. Clin. Imaging.

[10] Caruso D., Guido G., Zerunian M., Polidori T., Lucertini E., Pucciarelli F., Polici M., Rucci C., Bracci B., Nicolai M. (2021). Postacute sequelae of COVID-19 pneumonia: 6-month chest CT follow-up. Radiology.

[11] Han X., Fan Y., Alwalid O., Li N., Jia X., Yuan M., Li Y., Cao Y., Gu J., Wu H. (2021). Six-month follow-up chest CT findings after severe COVID-19 pneumonia. Radiology.

[12] Ribeiro Carvalho C.R., Lamas C.A., Chate R.C., Salge J.M., Sawamura M.V.Y., de Albuquerque A.L.P., Junior C.T., Lima D.M., Garcia M.L., Scudeller P.G. (2023). Long-term respiratory follow-up of ICU hospitalized COVID-19 patients: Prospective cohort study. PLoS ONE.

[13] Zapol W.M., Trelstad R.L., Coffey J.W., Tsai I., Salvador R.A. (1979). Pulmonary fibrosis in severe acute respiratory failure. Am. Rev. Respir. Dis..

[14] Madotto F., McNicholas B., Rezoagli E., Pham T., Laffey J.G., Bellani G., ESICM Trials Group (2021). Death in hospital following ICU discharge: Insights from the LUNG SAFE study. Crit. Care.

[15] Salehi S., Abedi A., Balakrishnan S., Gholamreza-Nezhad A. (2020). Coronavirus disease 2019 (COVID-19): A systematic review of imaging findings in 919 patients. Am. J. Roentgenol..

[16] Giovannetti G., De Michele L., De Ceglie M., Pierucci P., Mirabile A., Vita M., Palmieri V.O., Carpagnano G.E., Scardapane A., D’Agostino C. (2021). Lung ultrasonography for long-term follow-up of COVID-19 survivors compared to chest CT scan. Respir. Med..

[17] Ökmen K., Yildiz D.K., Soyaslan E., Ceylan I., Sayan H.E., Aytünür C.S. (2022). Comparison of two different lung ultrasound imaging protocols in COVID-19 pneumonia. Ultrasonography.

[18] Zhou B., Yang X., Zhang X., Curran W.J., Liu T. (2020). Ultrasound Elastography for Lung Disease Assessmalest. IEEE Trans. Ultrason. Ferroelectr. Freq. Control.

[19] Ramos Hernández C., Tilve Gomez A., Sánchez Fernández A., Cordovilla R., Núñez Ares A., Ordoñez Gómez P., Pérez A.W., Anón O.C., Ramírez J.G., Salas M.V. (2023). Multicentre study on the accuracy of lung ultrasound in the diagnosis and monitoring of respiratory sequelae in the medium and long term in patients with COVID-19. Front. Med..

[20] Zhang X., Zhou B., Osborn T., Bartholmai B., Kalra S. (2019). Lung ultrasound surface wave elastography for assessing interstitial lung disease. IEEE Trans. Biomed. Eng..

[21] Cooper B.G. (2011). An update on contraindications for lung function testing. Thorax.

[22] Hansell D.M., Bankier A.A., MacMahon H., McLoud T.C., Müller N.L., Remy J. (2008). Fleischner Society: Glossary of terms for thoracic imaging. Radiology.

[23] Hasan A.A., Maklouf H.A. (2014). B-lines: Transthoracic chest ultrasound signs useful in assessmalest of interstitial lung disease. Ann. Thorac. Med..

[24] Graham B.L., Steenbruggen I., Miller M.R., Barjaktarevic I.Z., Cooper B.G., Hall G.L., Hallstrand T.S., Kaminsky D.A., McCarthy K., McCormack M.C. (2019). Standardization of Spirometry 2019 Update. An Official American Thoracic Society and European Respiratory Society Technical Statemalest. Am. J. Respir. Crit. Care Med..

[25] Cohen J. (1992). Statistical Power Analysis. Curr. Dir. Psychol. Sci..

[26] Lorent N., Vande Weygaerde Y., Claeys E., Caamano Fajardo I.G., De Vos N., De Wever W., Salhi B., Gyselinck I., Bosteels C., Lambrecht B.N. (2022). Prospective longitudinal evaluation of hospitalized COVID-19 survivors 3 and 12 months after discharge. ERJ Open Res..

[27] Vexler A., Polyansky I., Gorodetsky R. (1999). Evaluation of skin viscoelasticity and anisotropy by measurement of speed of shear wave propagation with viscoelasticity skin analyser. J. Investig. Dermatol..

[28] Stumpf S., Jaeger H., Graeter T., Oeztuerk S., Schmidberger J., Haenleet M., Kratzer W., The Elasto-Study Group Ulm (2016). Influence of age, sex, body mass index, alcohol, and smoking on shear wave velocity (p-SWE) of the pancreas. Abdom. Radiol..

[29] Rossi A., Ganassini A., Tantucci C., Grassi V. (1996). Aging and the respiratory system. Aging.

[30] Dyer C. (2012). The interaction of ageing and lung disease. Chron. Respir. Dis..

[31] Enomoto T., Usuki J., Azuma A., Nakagawa T., Kudoh S. (2003). Diabetes mellitus may increase risk for idiopathic pulmonary fibrosis. Chest.

[32] Yang J., Xue Q., Miao L., Cai L. (2011). Pulmonary fibrosis: A possible diabetic complication. Diabetes Metab. Res. Rev..

[33] Wendel-Garcia P.D., Moser A., Jeitziner M.M., Aguirre-Bermeo H., Arias-Sanchez P., Apolo J., Roche-Campo F., Franch-Llasat D., Kleger G.R., Schrag C. (2022). Dynamics of disease characteristics and clinical management of critically ill COVID-19 patients over the time course of the pandemic: An analysis of the prospective, international, multicentre RISC-19-ICU registry. Crit. Care..

[34] Russo G., Flor N., Casella F., Ippolito S., Leidi F., Casazza G., Radovanovic D., Vezzulli F., Santus P., Cogliati C. (2022). Lung ultrasound in the follow-up of severe COVID-19 pneumonia: Six months evaluation and comparison with CT. Intern. Emerg. Med..

[35] Barbieri G., Gargani L., Lepri V., Spinelli S., Romei C., De Liperi A., Chimera D., Pistelli F., Carrozzi L., Corradi F. (2023). Long-term lung ultrasound follow-up in patients after COVID-19 pneumonia hospitalization: A prospective comparative study with chest computed tomography. Eur. J. Intern. Med..

[36] Dawod Y.T., Cook N.E., Graham W.B., Madhani-Lovely F., Thao C. (2020). Smoking-associated interstitial lung disease: Update and review. Expert. Rev. Respir. Med..

